# Letter to the Editor: Balancing scientific vigilance and psychological impact in the management of new COVID-19 variants

**DOI:** 10.1097/JS9.0000000000001733

**Published:** 2024-05-29

**Authors:** Shidong Wang, Dan Shan

**Affiliations:** a Department of Respiratory Medicine, Shaoxing Second Hospital, Zhejiang, People’s Republic of China; b Clinical Science Institute, University Hospital Galway, Galway, Ireland

*Dear Editor,*

The recent identification of the SARS-CoV-2 omicron subvariant BA.2.86, first detected in Denmark and known informally as ‘Pirola,’ has quickly captured significant attention from both the global scientific community and the general public, particularly due to its rapid spread compared to earlier variants^1^. We commend the work of Satapathy *et al*.^1^, as presented in their recent publication in your esteemed journal, entitled ‘The emergence and implications of SARS-CoV-2 omicron subvariant BA.2.86 on global health’. This study highlights the crucial roles of continuous genomic surveillance, global collaboration, adaptable response strategies, and sustained research efforts in managing such variants^1^. However, while the scientific focus on these new variants is justified, it is also vital to consider a comprehensive public health strategy, particularly in managing public perception to prevent undue panic and misinformation.

Historically, the emergence of new COVID-19 variants, such as the initial Omicron variant, has led to significant psychological and societal distress. This has been evidenced by increased anxiety and depression, particularly in regions experiencing surges, exacerbated by sensationalist media reporting^2^. These psychological impacts highlight the need for precise public health messaging to counteract misinformation and mitigate fear.

From an epidemiological perspective, the spread of BA.2.86 has been rapid but remains geographically limited, with only a modest number of cases detected across several countries^3^. Importantly, there have been very few, if any, reports of severe illness associated with BA.2.86 thus far^1^, suggesting that while vigilance is necessary, the variant may not pose a severe threat to global health in terms of critical disease outcomes at this stage. Furthermore, even though JN.1, the latest derivative of BA.2.86, has become the dominant variant in some regions such as the U.S., it has led to fewer medical interventions due to its lower severity despite higher infection rates^4^.

The psychological impacts of such variants can be profound and far-reaching. The uncertainty fuelled by memories of previous COVID-19 waves contributes to significant public anxiety^2^. It is crucial, therefore, that public health messages are crafted with clarity and precision to counter misinformation and alleviate unnecessary fears^5^. Health authorities, including governments and health organizations, should proactively engage with the public, focusing on the latest data concerning BA.2.86’s transmissibility, virulence, and the continued efficacy of current vaccines and treatments, to manage the psychological fallout of the pandemic without sensationalism. These updates should emphasize the adaptive capacity of public health responses and the adjustments being made in vaccine strategies and antiviral treatments to manage such variants. The latest and optimal recommendation for combatting new variants of COVID-19 remains vaccination. Although the most recent COVID-19 vaccine may not consistently prevent infections from JN.1 or other circulating Omicron subvariants, it can still reduce the severity of the disease in those who become infected^4^.

In addition to scientific and epidemiological responses, there is a critical need to enhance mental health services. The pandemic has underlined the essential role of mental health in overall health security. Strengthening resources for mental health support can help address the secondary effects of the pandemic, such as increased anxiety and other mental health disorders^2^. Collaborative efforts between public health authorities and mental health professionals are essential to provide clear, supportive guidance and interventions for those affected by pandemic-related stress.

In conclusion, while the detection of BA.2.86 warrants close scientific monitoring and may require strategic adjustments in our pandemic response, it is equally important to manage its psychological and social impacts^2^. By maintaining transparent communication and enhancing support systems, we can prevent undue public panic and ensure a balanced, health-oriented response to this evolving challenge. Moving forward, we strongly agree with *Satapathy et al.’s* perspective that more studies are necessary to understand the long-term impacts of the Omicron BA.2.86 subvariant and subsequent variants, and to ensure long-term preparedness for the largely unpredictable trajectory of the COVID-19 pandemic^1^.

## Ethical approval

Not applicable.

## Consent

Not applicable.

## Source of funding

Not applicable.

## Author contribution

S.W.: writing the manuscript draft; D.S.: study design and manuscript revision.

## Conflicts of interest disclosure

The authors declares no conflicts of interest.

## Research registration unique identifying number (UIN)

Not applicable.

## Guarantor

Dan Shan.

## Data availability statement

Not applicable.

## Provenance and peer review

Not invited.
